# Non-Invasive Remote Ischemic Preconditioning May Protect the Gastric Mucosa Against Ischemia-Reperfusion-Induced Injury Through Involvement of Glucocorticoids

**DOI:** 10.3389/fphar.2021.682643

**Published:** 2021-10-20

**Authors:** Ludmila Filaretova, Olga Komkova, Maria Sudalina, Natalia Yarushkina

**Affiliations:** Laboratory of Experimental Endocrinology, Pavlov Institute of Physiology, Russian Academy of Sciences, St. Petersburg, Russia

**Keywords:** gastric injury, ischemia-reperfusion, gastroprotection, remote ischemic preconditioning, glucocorticoids

## Abstract

Remote ischemic preconditioning (RIPC) is one of the most effective approaches to attenuate tissue injury caused by severe ischemia-reperfusion (I/R). Experimental studies have demonstrated that RIPC is capable of producing a protective effect not only on heart, but also on brain, lungs, kidneys, liver, intestine, and stomach. We previously demonstrated that glucocorticoids participate in protective effect of local gastric ischemic preconditioning against I/R-induced gastric injury. In the present study we investigated whether RIPC may protect the gastric mucosa against I/R-induced injury through involvement of glucocorticoids. Anesthetized fasted Sprague Dawley male rats were exposed to prolonged gastric I/R (30 min occlusion of celiac artery followed by 3 h of reperfusion) alone or with preliminary brief RIPC (10 min non-invasive occlusion of right hind limb blood flow followed by reperfusion for 30 min). First, we investigated the effect of RIPC on I/R-induced injury by itself. Then to study the role of glucocorticoids similar experiments were carried out: 1) in rats pretreated with the inhibitor of glucocorticoid synthesis, metyrapone (30 mg/kg, i.p), and in control animals; 2) in adrenalectomized rats without or with corticosterone replacement (4 mg/kg, s.c.) and in sham-operated animals; 3) in rats pretreated with glucocorticoid receptor antagonist RU-38486 (20 mg/kg, s.c.) and in control animals. I/R induced corticosterone rise and resulted in the gastric erosion formation. RIPC significantly reduced the erosion area in control animals. Metyrapone injected shortly before RIPC caused a decrease in plasma corticosterone levels and prevented the gastroprotective effect of RIPC and, moreover, further aggravated the deleterious effect of I/R. Adrenalectomy performed 1 week before experiment created long-lasting corticosterone deficiency and had no effect on the gastroprotective effect of RIPC. Nevertheless, corticosterone replacement which mimics the corticosterone rise, similar to RIPS, significantly reduced erosion areas of gastric mucosa in adrenalectomized rats supporting the role of glucocorticoids in gastroprotection. RU-38486, which occupied glucocorticoid receptors, similar to metyrapone prevented the gastroprotective effect of RIPC and, moreover, further aggravated the deleterious effect of I/R. The results of the present study demonstrate for the first time that RIPC may protect the gastric mucosa against I/R-induced injury through involvement of glucocorticoids.

## Introduction

Ischemic preconditioning (IPC) is an adaptive phenomenon, in which a tissue becomes more resistant to prolonged exposure of ischemia or ischemia reperfusion (I/R) after one or more brief preconditioning ischemia-reperfusion episodes (Aggarwal et al., 2016; McDonough and Weinstein, 2020). The phenomenon of IPC was first depicted as early as 1986 by Murry et al. (1986). It has been shown in dogs that multiple brief ischemic episodes protect the heart from a subsequent sustained ischemic insult (Murry et al., 1986). Later a similar model of ischemic tolerance was also described in rat, rabbit and pig hearts (Kloner and Jennings, 2001). The concept of IPC was supported in humans (Kloner and Yellon, 1994).

Although early IPC studies were related with the heart, the protective effects IPC has been demonstrated in multiple organ systems and shown to be effective. Various organs including kidneys (Fuller et al., 2005), lungs (Soncul et al., 1999), liver (Fernández et al., 2002), stomach (Pajdo et al., 2001) or brain (Li et al., 2017) also respond to brief ischemia exposures with an increased resistance to severe ischemia.

Initially the effect of IPC was assumed to implement only within the tissue subjected to prolonged ischemia and, in this case, IPC is considered as local or regional phenomenon. But in 1993 Karin Przyklenk with colleagues showed in canine model that brief episodes of ischemia in one vascular bed may protect remote myocardium from subsequent sustained coronary artery occlusion (Przyklenk et al., 1993). This phenomenon was termed as remote ischemic preconditioning (RIPC) (Przyklenk et al., 1993). There is growing experimental and clinical evidence supporting that RIPS elicits cardioprotection. It is considered as the most practical, non-invasive, cost-free, and clinically compatible, secure procedure for reducing I/R-induced injury. The use of a conventional blood pressure cuff on the upper or lower limb in eliciting cardioprotection has expedited its clinical applicability (Uutela et al., 2020).

After discovery by Karin Przyklenk RIPC phenomenon regarding heart, it was demonstrated that RIPC similar to local IPC is common physiological phenomenon. Indeed, experimental studies found that it was possible to protect non-cardiac organs and tissues from acute I/R injury (Lim and Hausenloy, 2012; Wang et al., 2015).

The findings suggest that RIPC represents a form of systemic protection against acute I/R injury and may provide non-invasive endogenous therapeutics strategy for protection organs against acute I/R (Lim and Hausenloy, 2012; Heusch and Gersh, 2016). RIPC and the mechanisms underlying this phenomenon have been extensively studied in animal models and cardiac surgery, as well as in solid organ transplantation (Uutela et al., 2020). Three main pathways for transmitting the protective signal from the organ or tissue, in which the RIPC stimulus is applied, to the target organ or tissue, are considered: neural pathway, the release of circulating humoral factors (humoral pathway) and activation of a systemic protective effect (systemic response) (Lim and Hausenloy, 2012).

Injury caused by ischemia or I/R plays a significant role in the abdominal organ diseases, in the gastric mucosal injury in particular, and development new approaches to attenuate or prevent the pathology is extremely important. RIPC is one of the most effective non-invasive approaches to attenuate tissue gastric injury caused by severe I/R. RIPC-induced gastroprotection was first demonstrated by Brzozowski et al. (2004a) using a rat model of gastric I/R-caused injury. The authors showed that RIPC applied to the heart or liver significantly improved gastric blood flow and reduced gastric injury via the mechanisms involving prostaglandin E2 (PGs) production and the activation of sensory nerves releasing calcitonin gene-related peptide (CGRP) combined with the suppression of plasma proinflammatory cytokines (IL-1β and TNFα) levels (Brzozowski et al., 2004a; Brozowski et al., 2004b).

No study so far has been undertaken to verify whether glucocorticoids may participate in realization of protective effect of RIPC against I/R-induced gastric injury. Glucocorticoids are key hormonal factors providing systemic response to stressor. Taking into consideration this fact together with the facts that humoral pathway and systemic response are two main pathways for the transmitting the RIPC-induced protective signals (Lim and Hausenloy, 2012) it is reasonable to propose a participation of glucocorticoids in realization of gastroprotective effect of RIPC. Our previous findings about gastroprotective role of glucocorticoids also allowed us to assume that glucocorticoids may contribute to gastroprotective effect of RIPC (Filaretova et al., 1998; Filaretova et al., 2004; Filaretova et al., 2007; Filaretova et al., 2008, Filaretova et al., 2012; Filaretova, 2011; Filaretova, 2017; Filaretova and Bagaeva, 2016). Additionally it is important to note that we previously demonstrated participation of glucocorticoids in protective effect of local gastric ischemic preconditioning against I/R-induced gastric injury (Bobryshev et al., 2009).

In the present study we investigated whether RIPC may protect the gastric mucosa against IR-induced injury through involvement of glucocorticoids.

## Materials and Methods

### Animals

Male Sprague-Dawley rats (Stolbovoe, Moscow, Russia) weighing about 250–300 g were housed six animals per cage and have been acclimated to the standard laboratory conditions (12:12-h light-dark cycle, temperature 20 ± 1°C, free access to food and water) for a week. The animals were fasted 24 h before the experiment but given free access to drinking water. The care and treatment of animals were done in accordance with ARRIVE guidelines and EU Directive 2010/63/EU for animal experiments and was reviewed and approved by the Institutional Animal Care and Use Committee of the Pavlov Institute of Physiology RAS.

### Production of Gastric Lesions Induced by Ischemia/Reperfusion

Gastric erosions were produced by prolonged I/R in preliminary (24 h) fasted anesthetized rats. We used a well known experimental model of I/R to produce gastric erosions (Wada et al., 1996). Animals were given with tiletamine/zolazepam (Zoletil ®, «Virbac», Carros, France, 7.5 mg/kg, ip) and xylazine hydrochloride (Rometar, «SPOFA», Luberec, Czech Republic, 10 mg/kg, ip) in a volume of 0.5 ml/kg for general anaesthesia. Additionally, Zoletil (4 mg/kg) was administered every 40 min to maintain an adequate level of anesthesia. Under anesthesia, the abdomen was opened, the celiac artery clamped with a small vascular clamp for 30 min followed by removal of this clamp to obtain reperfusion for 3 h (in total 3.5 h). Sham-operated animals subjected to the same surgical procedure but the celiac artery was not occluded. After completion of 3.5 h I/R, anesthetized animals were euthanized, and the stomachs were removed for examination of gastric lesions (the area in mm 2). The area of lesions developed in the corpus mucosa was measured using computer program ImageJ, summed per stomach, and used as a lesion score.

### Remote Ischemic Preconditioning

In our study we used hind limb ischemic preconditioning as RIPC (Souza Filho et al., 2009). RIPC included 10 min non-invasive occlusion of right hind limb blood flow followed by reperfusion for 30 min (in total 40 min). Non-invasive occlusion of hind limb blood flow was produced by placing a forceps to the quadriceps femoris muscle; then, they were removed for reperfusion.

### Approaches

**FIGURE 1:**
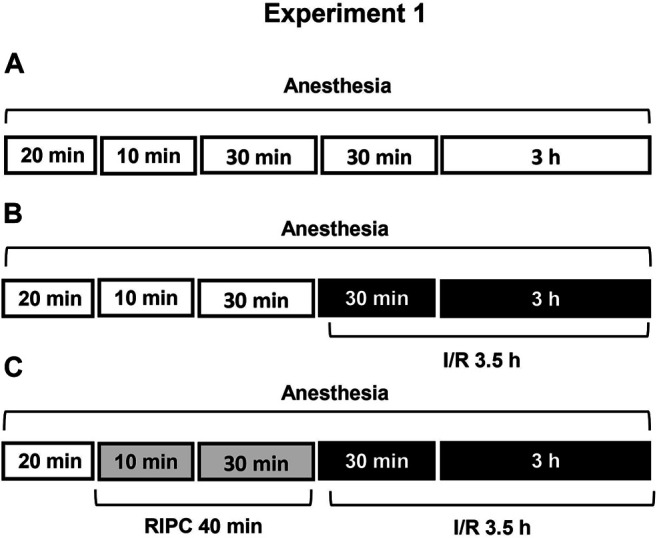
Design of Experiment 1: effect of RIPC on I/R-induced gastric erosions and plasma corticosterone levels. Three groups of rats were used: control rats without any exposures (I/R or/and RIPC) **(A)**; rats which were subjected I/R alone **(B)** or preliminary RIPC before I/R **(C)**.

**FIGURE 2:**
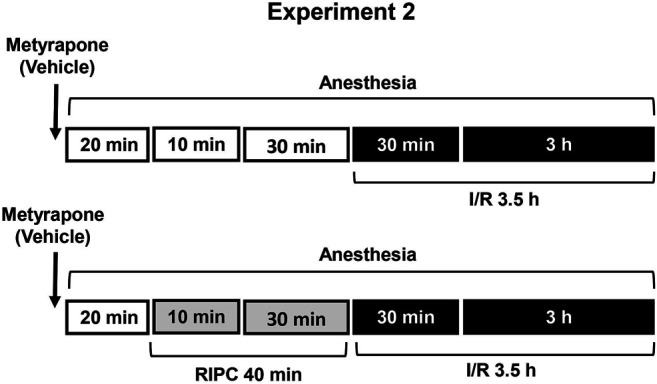
Design of Experiment 2: effect of metyrapone pretreatment on gastroprotective action of RIPC and plasma corticosterone levels. Four groups of rats were used: rats pretreated with metyrapone (30 mg/kg, i.p.) or its vehicle which were subjected to I/R alone and rats pretreated with metyrapone or its vehicle which were subjected to preliminary RIPC before I/R.

**FIGURE 3:**
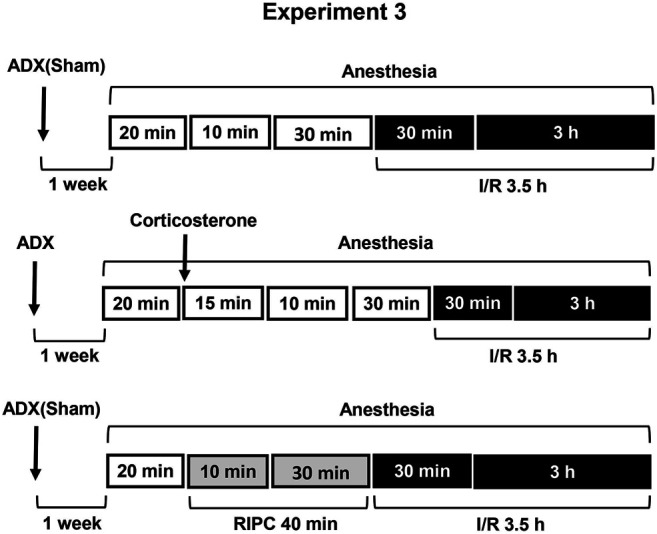
Design of Experiment 3: effect of adrenalectomy and corticosterone replacement on gastroprotective action of RIPC and plasma corticosterone levels. Adrenalectomized and sham-operated groups of rats were subjected to I/R alone or preliminary RIPC before I/R. One group of adrenalectomized rats was administered by corticosterone (4 mg/kg, s.c.), instead of RIPC, before I/R to mimic corticosterone rise induced by RIPC.

**FIGURE 4:**
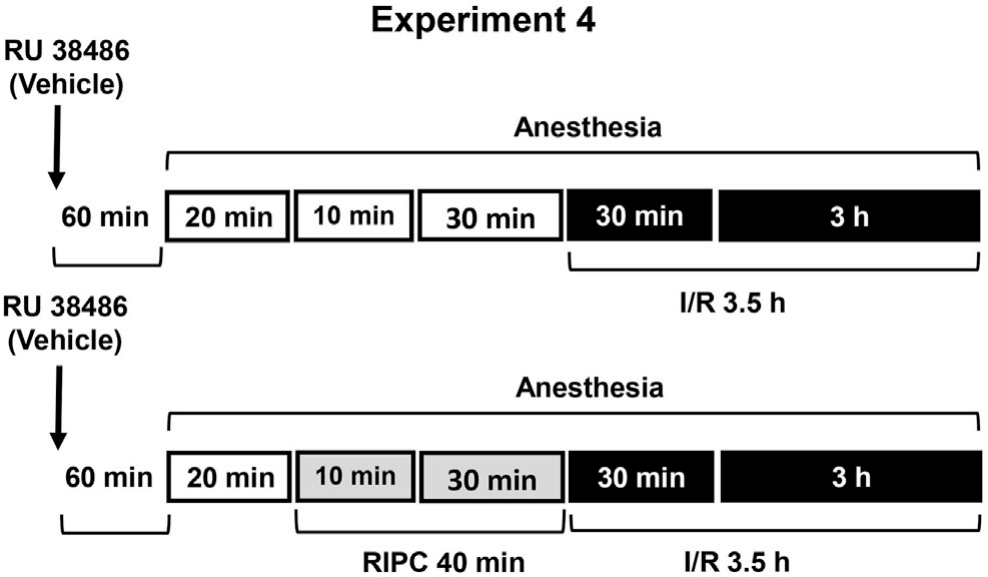
Design of Experiment 4: effect of RU-38486 pretreatment on gastroprotective action of RIPC and plasma corticosterone levels. Four groups of rats were used: rats pretreated with RU-38486 (20 mg/kg, s.c.) or its vehicle which were subjected to I/R alone and rats pretreated with RU-38486 or its vehicle which were subjected to preliminary RIPC before I/R.

**FIGURE 5:**
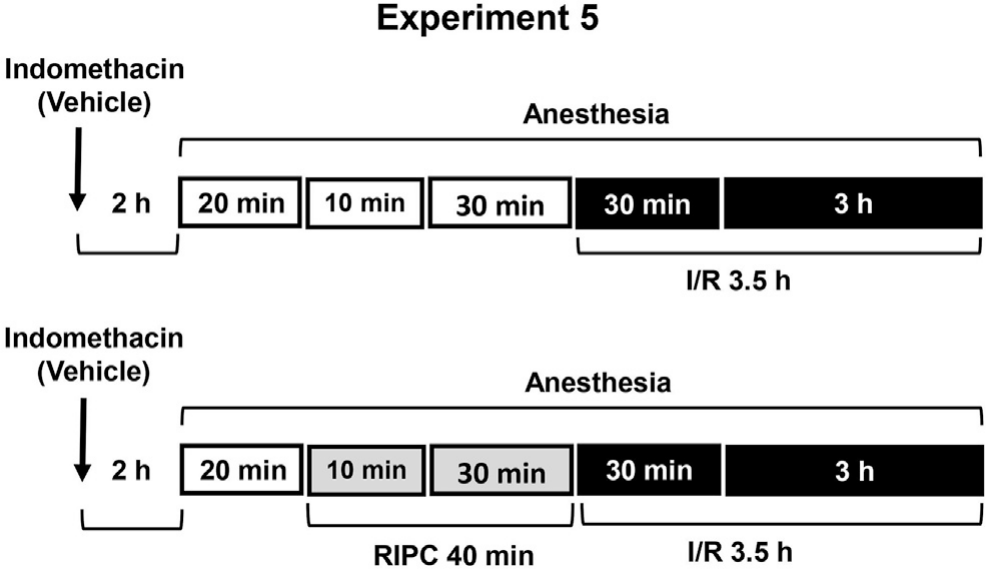
Design of Experiment 5: effect of indomethacin on gastroprotective action of RIPC and plasma corticosterone levels. Four groups of rats were used: rats pretreated with indomethacin (5 mg/kg, s.c.) or its vehicle which were subjected to I/R alone and rats pretreated with indomethacin or its vehicle which were subjected to preliminary RIPC before I/R.

First, we examined the protective effect of RIPC against I/R-induced gastric erosion formation (Experiment 1) (Figure 1). To verify whether glucocorticoids contribute to protective effect of RIPC against I/R-induced gastric injury we compared the effects of RIPC in rats with normal and deficient corticosterone production as well as in rats with normal and occupied glucocorticoid receptors. Glucocorticoid deficiency was created by inhibition of glucocorticoid synthesis by metyrapone (Experiment 2) **(** Figure 2) or adrenalectomy (Experiment 3) **(** Figure 3). Corticosterone replacement was used to mimic corticosterone rise in adrenalectomized rats (Experiment 3) **(** Figure 3). The antagonist of glucocorticoid receptors RU-38486 was used for occupation of glucocorticoid receptors (Experiment 4) **(** Figure 4). To estimate the contribution of prostaglandins to gastroprotective action of RIPC, we compared the effects of RIPC against the I/R-induced gastric erosion in rats with normal and deficient prostaglandin production (Experiment 5) **(** Figure 5). Deficiency of prostaglandins was created by non-selective inhibitor of cyclooxygenase 1 and 2 indomethacin administered at the non-ulcerogenic dose.

Experiment 1 Effect of RIPC on I/R-Induced Gastric Erosions and Plasma Corticosterone Levels.

Figure 1 is showing design of experiment. Twenty minutes after the onset of anesthesia, fasted rats were exposed to prolonged gastric I/R (30 min occlusion of celiac artery followed by 3 h of reperfusion) alone or with preliminary brief RIPC (10 min non-invasive occlusion of right hind limb blood flow followed by reperfusion for 30 min). Control animals were anesthetized and kept at the same conditions, but did not subject any surgery or RIPC.

Experiment 2 Effect of Metyrapone Pretreatment on Gastroprotective Action of RIPC and Plasma Corticosterone Levels.

Metyrapone (Sigma-Aldrich, Steinheim, Germany), inhibitor of glucocorticoid synthesis, was injected at the dose of 30 mg/kg (in 5 ml/kg 0.9% normal saline with drop of Tween-80, i.p.) 20 min before RIPC **(** Figure 2 **)**. Control rats were received vehicle of metyrapone. Immediately after metyrapone or vehicle injection all animals were anesthetized. Twenty minutes later one half each group was exposed to RIPC for 40 min, other half each group kept at the same conditions, but without RIPC. Then, all animals were exposed to I/R for 3.5 h. After completion of I/R anesthetized animals were decapitated.

Experiment 3 Effect of Adrenalectomy and Corticosterone Replacement on Gastroprotective Action of RIPC and Plasma Corticosterone Levels.

Adrenalectomy as well as sham operation was performed 1 week before experiment under ether anesthesia. Sham-operated rats were subjected to the same surgical procedure, but the adrenals were not removed (Figure 3). After surgery adrenalectomized rats were provided with a 0.9% NaCl solution in addition to tap water in the home cage. Corticosterone replacement was performed by injecting corticosterone (Sigma, Steinheim, Germany, 4 mg/kg in 1 ml/kg 1,2-propylene glycol s.c.) to adrenalectomized rats 55 min before I/R.

The experiment was carried out in sham-operated and adrenalectomized rats: one half each group was exposed to RIPC for 40 min, other half each group kept at the same conditions, but without RIPC. Then, all animals were exposed to I/R for 3.5 h and decapitated after completion of I/R.

Experiment 4 Effect of RU-38486 Pretreatment on Gastroprotective Action of RIPC and Plasma Corticosterone Levels.

The antagonist of glucocorticoid receptors RU-38486 (Sigma, Saint Louis, United States) was injected at the dose of 20 mg/kg (in 5 ml/kg 1,2-propylene glycol, s.c.) 1 h 20 min before RIPC **(** Figure 4 **)**. Control rats were received vehicle of RU-38486. The experiment was carried out in rats pretreated with RU-38486 or its vehicle. One half each group was exposed to RIPC for 40 min, other half each group kept at the same conditions, but without RIPC. Then, all animals were exposed to I/R for 3.5 h and decapitated after completion of I/R.

Experiment 5 Effect of Indomethacin on Gastroprotective Action of RIPC and Plasma Corticosterone Levels.

To evaluate the contribution of prostaglandins, non-selective inhibitor of cyclooxygenase 1 and 2 indomethacin at the non-ulcerogenic dose (5 mg/kg, in 5 ml/kg 0.9% normal saline with drop of Tween-80, s.c.) was administered 2 h 20 min before RIPC **(** Figure 5 **)**. Control rats were received vehicle of indomethacin. The experiment was carried out in rats pretreated with indomethacin or its vehicle: one half each group was exposed to RIPC for 40 min, other half each group kept at the same conditions, but without RIPC. Then, all animals were exposed to I/R for 3.5 h and decapitated after completion of I/R.

### Collection of Blood Samples and Determination of Plasma Corticosterone Levels

Blood samples were collected from trunk vessels after decapitation. Plasma samples were obtained by blood centrifugation at 3,000 revolutions/min, for 15 min at 4°C. Samples were stored at −20°C until further analysis. The concentration of corticosterone in plasma was determined using commercial ELISA kits (K210R, “HEMA”, Russia). Detection level was 5 nmol/L according to the manufacturer.

### Statistical Analysis

Data was expressed as mean ± SEM. Data was analyzed with ANOVA module of the МеdCаlс Version 12.7.0.0. (Statictics for biomedical research, MedCalc Software, Belgium). Statistical significances were tested by one way ANOVA followed by a post hoc Student-Neuman-Keuls. Levene’s test was applied before post hoc test for validation of the equality of variances. In case of different variances Kruskal-Wallis test was used. In each case, the required level for significance was considered to be *p* < 0.05.

## Results

### Effect of Remote Ischemic Preconditioning on Plasma Corticosterone Levels and Gastric Erosion Induced by Ischemia-Reperfusion

**FIGURE 6:**
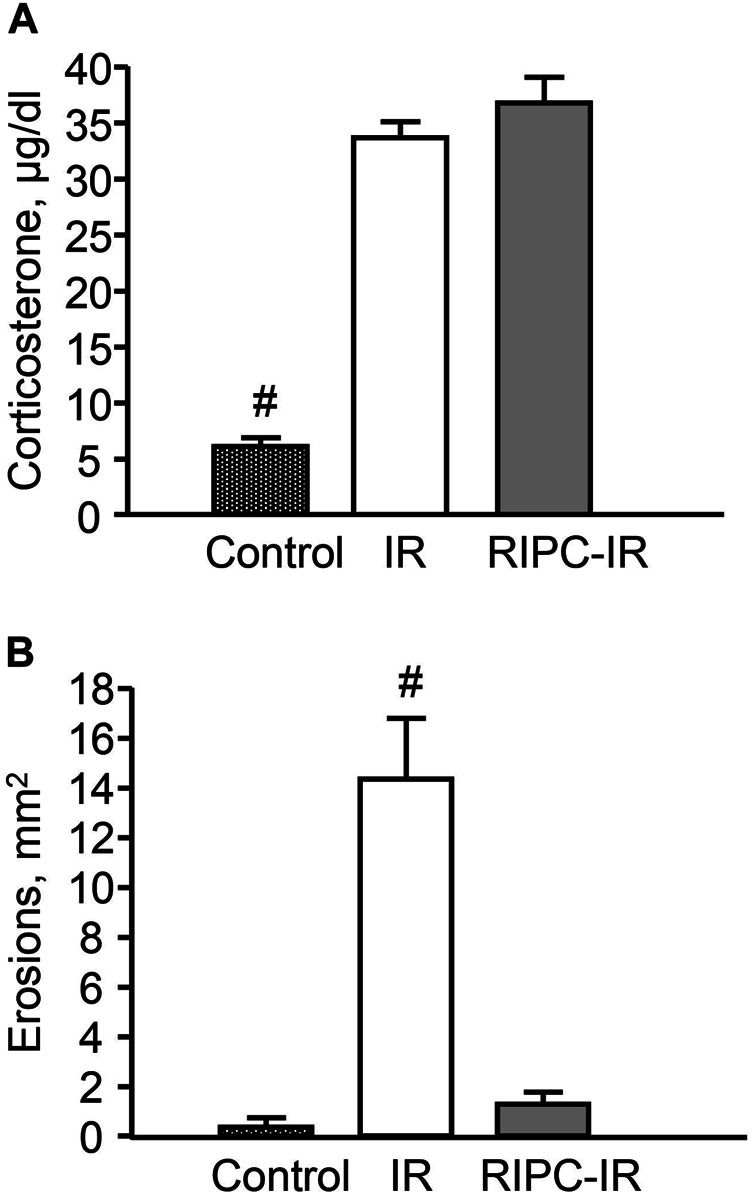
Effect of remote ischemic preconditioning (RIPC) on ischemia-reperfusion (I/R)-induced plasma corticosterone levels (**A)** and gastric erosions (**B**) in rats. Significant differences at *p* < 0.05; # from all groups; number of rats per group (*n*): Control (n = 6); I/R (*n* = 10); RIPC-I/R (*n* = 11).

I/R significantly (*p* < 0.05) increased plasma corticosterone level as compared to control animals (Figure 6A) [H (2; 27) = 13,9263; *p* = 0.0009]. RIPC did not further influence on I/R-induced corticosterone rise: there were no significant differences in I/R-induced corticosterone levels between the groups subjected to I/R alone (without RIPC) and with preliminary RIPC (Figure 6A).

Exposure to I/R for 3.5 h produced gastric erosion in preliminary fasted rats. The hemorrhagic erosion occurred mostly in the corpus mucosa. RIPC significantly (*p* < 0.05) reduced the I/R-induced gastric erosion (gastroprotective effect) [H (2; 27) = 17,5574; *p* = 0.0002] (Figure 6B).

There were no gastric erosion in the pre-starved control animals without I/R or/and RIPC (Figure 6B).

### Effect of Pretreatment With Metyrapone on Gastroprotective Action of Remote Ischemic Preconditioning

**FIGURE 7:**
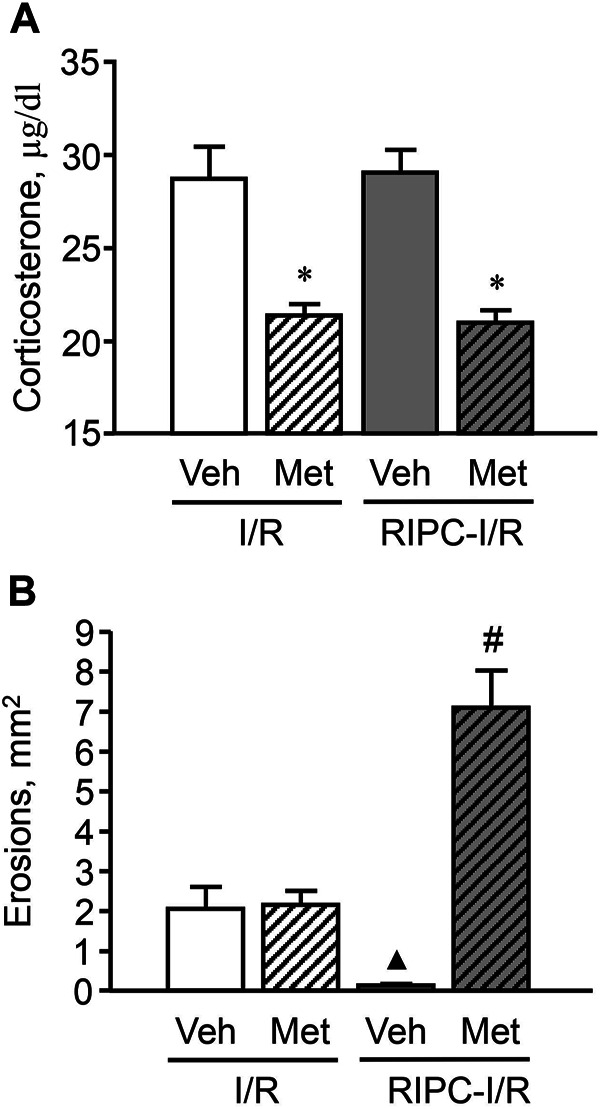
Effect of pretreatment with metyrapone on ischemia-reperfusion (I/R)-induced plasma corticosterone levels **(A)** and gastric erosions **(B)** in rats with and without remote ischemic preconditioning (RIPC). Significant differences at *p* < 0.05; * vs. corresponding vehicle (Veh) group; ▲ vs. “Veh + I/R” group; # from all groups; number of rats per group (n): Veh + I/R (*n* = 11); Met + I/R (*n* = 16); Veh + RIPC-I/R (*n* = 15); Met + RIPC-I/R (*n* = 16).

In rats pretreated with vehicle of metyrapone **(** Figure 7A) I/R-induced plasma corticosterone levels were as high as corticosterone levels in rats treated with I/R alone (Figure 6A **)**. RIPC did not change plasma corticosterone levels in vehicle-treated rats (Figure 7A). Metyrapone injected shortly before RIPC caused a decrease (*p* < 0.05) of corticosterone response to I/R (Figure 7A) in both rats with and without RIPC [H (3; 58) = 31,1791; *p* = 0,0001].

Metyrapone had no effect on I/R-induced gastric injury by itself. However, it prevented the gastroprotective effect of RIPC. Moreover, further aggravated the deleterious effect of I/R (Figure 7B). Indeed, the average area of gastric lesion caused by I/R in metyrapone-pretreated animals with RIPC was significantly greater (*p* < 0.05) than that in the vehicle- and metyrapone-pretreated groups without RIPC [H (3; 58) = 32,7465; *p* = 0.0001].

### Effect of Adrenalectomy on Gastroprotective Action of Remote Ischemic Preconditioning

**FIGURE 8:**
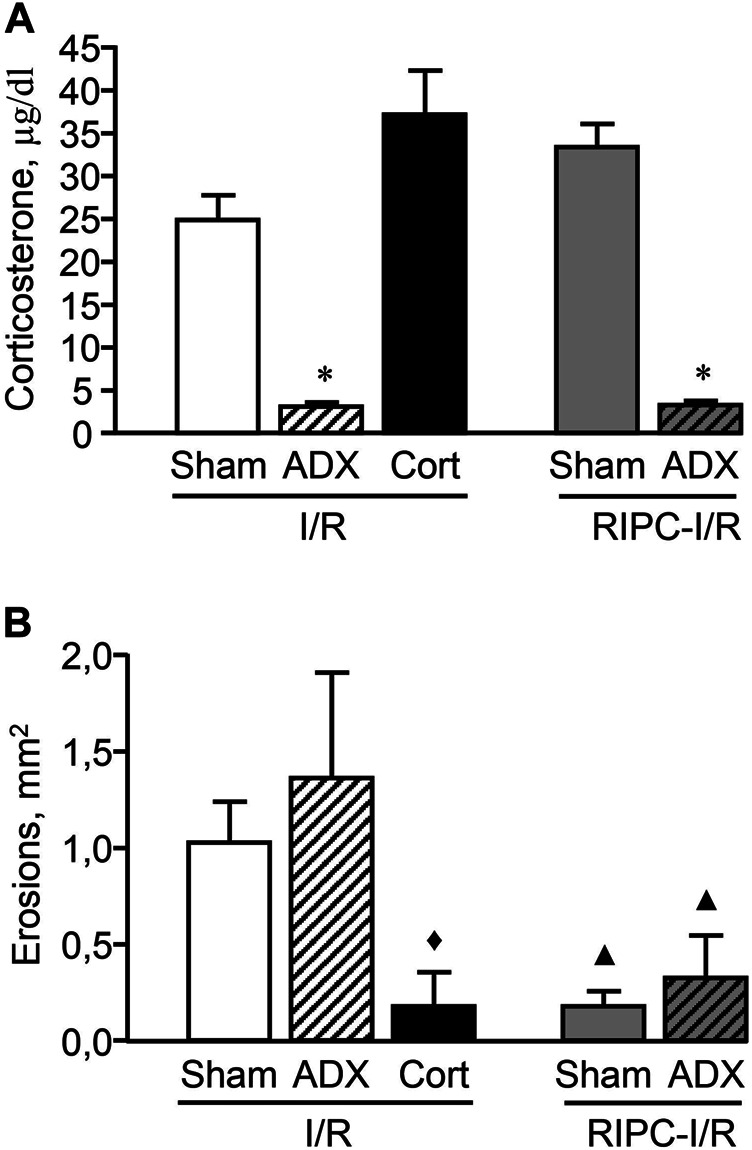
Effect of adrenalectomy and corticosterone replacement on ischemia-reperfusion (I/R)-induced plasma corticosterone levels (**A**) and gastric erosions (**B**) in rats with and without remote ischemic preconditioning (RIPC). Significant differences at *p* < 0.05; * vs sham-operated (Sham) group (A); ▲ vs. corresponding Sham or ADX (adrenalectomy) groups in rats without RIPC; ♦ vs. “ADX + I/R” group (B); Cort–corticosterone replacement; number of rats per group (n): Sham + I/R (*n* = 11); ADX + I/R (*n* = 10); Cort + I/R (*n* = 7); Sham + RIPC-I/R (*n* = 8); ADX + RIPC-I/R (*n* = 9).

Adrenalectomy performed 1 week before experiment created long-lasting corticosterone deficiency (Figure 8A). The adrenalectomized rats subjected I/R alone as well as with preliminary RIPC had lower (*p* < 0.05) plasma corticosterone levels compared to sham-operated animals [H (4; 45) = 33,7527; *p* = 0.0001]. Corticosterone replacement mimicked I/R-induced corticosterone rise in adrenalectomized rats (Figure 8A). There were no differences in I/R-induced plasma corticosterone levels in adrenalectomized rats and sham-operated animals after corticosterone replacement.

Adrenalectomy had no effect on I/R-induced gastric injury as well as the gastroprotective effect of RIPC. RIPC attenuated (*p* < 0.05) I/R-induced gastric injury in sham-operated as well as adrenalectomized rats [H (4; 45) = 14,2686; *p* = 0.0065] (Figure 8B). Nevertheless, corticosterone replacement which mimics the corticosterone rise, similar to RIPS, significantly (*p* < 0.05) reduced erosion areas of gastric mucosa in adrenalectomized rats supporting the role of glucocorticoids in gastroprotection (Figure 8B).

### Effect of Pretreatment With RU-38486 on Gastroprotective Action of Remote Ischemic Preconditioning

**FIGURE 9:**
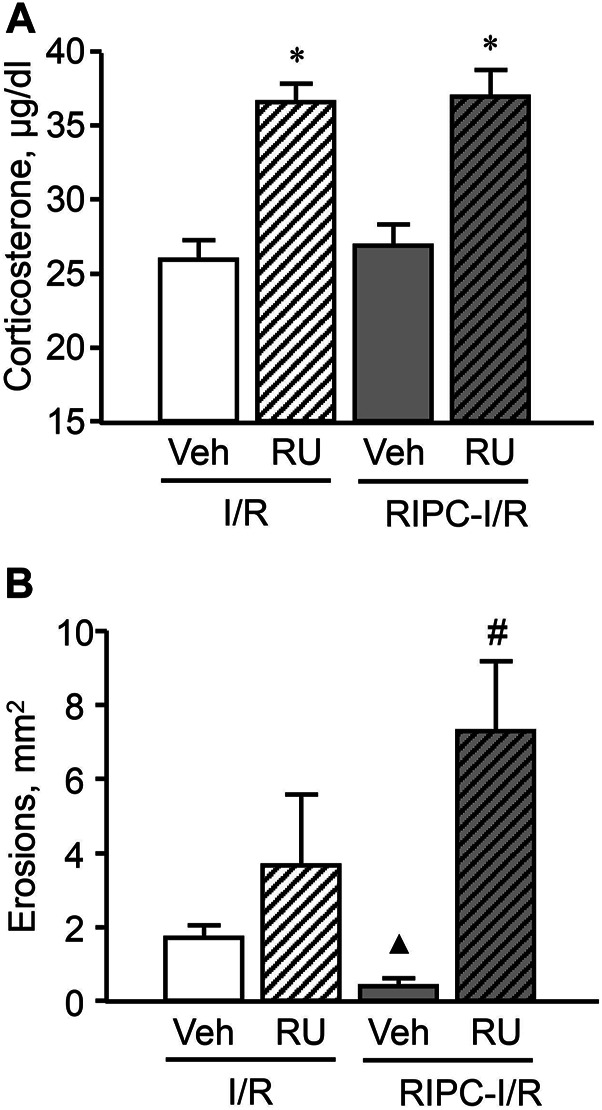
Effect of pretreatment with RU-38486 on ischemia-reperfusion (I/R)-induced plasma corticosterone levels (**A**) and gastric erosions (**B**) in rats with and without remote ischemic preconditioning (RIPC). Significant differences at *p* < 0.05; * vs. vehicle (Veh) group; ▲ vs. “Veh + IR” group; # from all groups; number of rats per group (n): Veh + I/R (*n* = 14); RU + I/R (*n* = 12); Veh + RIPC-I/R (*n* = 14); RU + RIPC-I/R (*n* = 12).

Pretreatment with the antagonist of glucocorticoid receptors RU-38486 potentiated an increase of the I/R-induced corticosterone level in rats with as well as without RIPC [F (3; 48) = 16,7952; *p* = 0.0001] (Figure 9A). The increased plasma corticosterone level in RU-38486-pretreated rats can be considered as an evidence of occupation of glucocorticoid receptors by their antagonist.

RU-38486, which occupied glucocorticoid receptors, had no effect on I/R-induced gastric injury by itself but similar to metyrapone prevented the gastroprotective effect of RIPC and, moreover, further aggravated the deleterious effect of IR (Figure 9B). Specifically, the mean area of I/R-induced gastric injury after RIPC was significantly (*p* < 0.05) greater in rats pretreated with RU-38486 than in rats pretreated with RU-38486 vehicle subjected I/R alone or with preliminary RIPC (H (3; 52) = 22,4574; *p* = 0,0001).

### Effect of Pretreatment With Indomethacin on the Gastroprotective Action of Remote Ischemic Preconditioning

**FIGURE 10:**
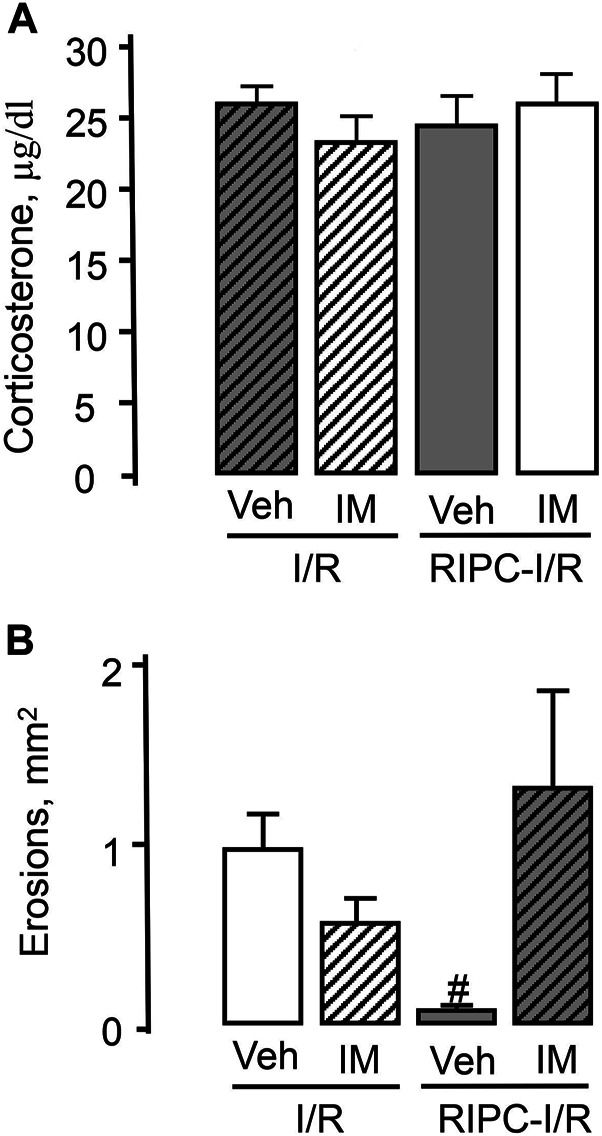
Effect of pretreatment with indomethacin on ischemia-reperfusion (I/R)-induced plasma corticosterone levels **(A)** and gastric erosions **(B)** in rats with and without remote ischemic preconditioning (RIPC).Significant differences at *p* < 0.05; # from all groups; number of rats per group (n): Veh + I/R (*n* = 27); IM + I/R (*n* = 24); Veh + RIPC-I/R (*n* = 12); IM + RIPC-I/R (*n* = 13).

Pretreatment with indomethacin at the non-ulcerogenic dose (5 mg/kg, s.c.) that suppressed mucosal generation of prostaglandins did not affect I/R-induced plasma corticosterone levels in rats subjected I/R alone or in combination with RIPC (Figure 10A).

Indomethacin had no significant effect on I/R-induced gastric injury by itself. However, indomethacin pretreatment prevented the gastroprotective effect of RIPC (Figure 10B). Indeed, RIPC reduced (*p* < 0.05) the area of I/R-induced gastric lesion in indomethacin vehicle pretreated rats, whereas in indomethacin-pretreated rats this area did not differ the area of I/R-induced gastric lesion without RIPC and was significantly (*p* < 0.05) greater compared to that in vehicle-treated rats with preliminary RIPC [H (3; 76) = 15.72; *p* = 0.001] (Figure 10B).

## Discussion

The results of the present study show that 3.5 h I/R, which resulted in the gastric erosion formation, markedly increased plasma corticosterone level in rats. RIPC significantly reduced the erosion area in control animals. The gastroprotective effect of RIPC was eliminated by the pretreatment rats with metyrapone, inhibitor glucocorticoid synthesis, as well as with RU-38486, glucocorticoid receptor antagonist. The data suggest that glucocorticoids may be involved in gastroprotective effect of RIPC against I/R-induced gastric injury.

Gastroprotective effect of RIPC demonstrated in our study is consistent with the data of literature (Brzozowski et al., 2004a), but it should be noted that in our study the effect was more pronounced.

A new knowledge revealed in this study is the fact of the involvement of glucocorticoids in the implementation of the gastroprotective effect of RIPC.

It is the first study aimed to verify whether glucocorticoids may participate in realization of protective effect of RIPC against IR-induced gastric injury. It was not assumed before to verify the participation of glucocorticoids in gastroprotective effect of RIPS, apparently due to the prevailing traditional point of view on ulcerogenic action of glucocorticoids released during stress. Nevertheless, our previous findings suggest that glucocorticoids released during acute stress-induced activation of the hypothalamic-pituitary-adrenocortical (HPA) axis are naturally occurring protective factors that play an important role in maintenance of the gastric mucosal integrity (Filaretova et al., 1998; Filaretova et al., 2012; Filaretova 2011; Filaretova and Bagaeva 2016). It was demonstrated that glucocorticoids released during preconditioning mild stress contribute to the protective effect of this stress on gastric mucosa against cold-restraint-induced gastric lesions (Filaretova et al., 2008; Filaretova, 2017). Moreover, we found that glucocorticoids produced in response to various ulcerogenic stimuli such as I/R, indomethacin, aspirin, ethanol, acetic acid also play gastroprotective role (Bobryshev et al., 2009; Filaretova 2011). Gastroprotective effects of glucocorticoids may be mediated by multiple actions, including maintenance of gastric mucosal blood flow, mucus production, and attenuation of enhanced gastric motility and microvascular permeability (Filaretova et al., 2004; Filaretova 2011). In addition, glucocorticoids released during activation of the HPA axis may contribute to protection of the gastric mucosa by maintaining general body homeostasis, including glucose levels and systemic blood pressure, which could be a basis for their beneficial influence on gastric mucosal integrity (Filaretova et al., 2004). Furthermore, glucocorticoids exert a compensatory gastroprotective role in the case of impaired gastroprotective mechanisms provided by PGs, nitric oxide (NO), and capsaicin-sensitive sensory neurons (Filaretova et al., 2007). Taking into consideration beneficial action of glucocorticoids on the gastric mucosa, gastric blood flow particularly, and general body homeostasis (Filaretova et al., 2004) first we supposed that glucocorticoids may contribute to gastroprotective effect of local ischemic preconditioning. The results obtained indeed confirmed that glucocorticoids participate in protective effect of gastric IPC against IR-induced gastric injury (Bobryshev et al., 2009; Filaretova 2017). In the present study we moved further demonstrating a participation of glucocorticoids in gastroprotective effect RIPC too. The present findings are additional strong support of gastroprotective nature of glucocorticoids.

To test the participation of glucocorticoids in RIPC-induced gastroprotection the three approaches were used in the present study.

Adrenalectomy has frequently been used for studying the role of glucocorticoids in gastric erosions, with conflicting results. Adrenalectomy has been used to understand effects of stress-produced glucocorticoids on the gastric mucosa and mechanisms of gastroprotective action of corticotropin-releasing factor (CRF) and results obtained in adrenalectomized rats (in cold-restrained ulcerogenic model) do not support gastroprotective role of glucocorticoids produced in stress as well as participation of glucocorticoids in gastroprotective action of CRF. Nevertheless, using several other approaches for creating glucocorticoid deficiency, not interfering with adrenomedullary catecholamines, we obtained convincing results about gastroprotective role glucocorticoids released during activation of HPA axis (Filaretova et al., 1998; Filaretova et al., 2004; Filaretova et al., 2008; Filaretova, 2011; Filaretova, 2017; Filaretova and Bagaeva, 2016) and their participation in gastroprotective action of CRF (Filaretova et al., 2012).

One reason for the inconsistent effects of adrenalectomy may be the removal of adrenomedullary catecholamines that can provoke acute gastric lesions in experimental animals and we discussed it (see Filaretova et al., 1998). Additionally to a lack of catecholamines, long-lasting glucocorticoid deficiency triggers systemic homeostatic shifts as well as protective processes including an increase in CRF production which may attenuate negative effects of glucocorticoid deficiency (Filaretova et al., 2012).

In the present study glucocorticoid deficiency caused by adrenalectomy led to an exacerbation of inflammation in gastric mucosa, but had no effect on the gastroprotective effect of RIPC. The excessive production of CRF caused by the lack of glucocorticoids within the HPA feedback loop (Uchoa et al., 2014) could be one of the reasons. Additional studies with CRF receptor antagonists are necessary to check this suggestion. Nevertheless, corticosterone replacement which mimics the corticosterone rise, similar to RIPS, significantly reduced erosion areas of gastric mucosa in adrenalectomized rats supporting the role of glucocorticoids in gastroprotection.

Pretreatment with metyrapone, the inhibitor of glucocorticoid synthesis was the most suitable approach because of a short-lasting inhibiting effect of the drug (Mikics et al., 2004). Metyrapone pretreatment allowed us to prevent the acute corticosterone response and avoid the long-lasting effects of glucocorticoid deficiency. Metyrapone injected shortly before RIPC caused a decrease in plasma corticosterone levels and prevented the gastroprotective effect of RIPC and, moreover, further aggravated the deleterious effect of I/R. The results support a participation of corticosterone in a realization of gastroprotective effect of RIPC in rats.

Further support for participation of glucocorticoids in the protective effects of RIPC against I/R-induced gastric injury comes from our experiments with pretreatment by glucocorticoid receptor antagonist RU-38486. Since the RU-38486 is a high affinity antagonist of glucocorticoid receptors and prevents their translocation to the nucleus, it was applied to interrupt genomic signaling pathways of glucocorticoids (Alexandrová, 1994; Bartholomew et al., 1997). A blockade of glucocorticoid receptors increases corticosterone level by activating negative feedback (Filaretova et al., 2002) and we observed this increase in our experiments as a marker of an occupation of glucocorticoid receptors by their antagonist. RIPC-caused gastroprotective effect was not observed in the rats pretreated by RU-38486. RU-38486 similar to metyrapone not only prevented the gastroprotective effect of RIPC but, moreover, further aggravated the deleterious effect of I/R. The results obtained using metyrapone and RU-38486 pretreatments taken together argue for the participation of glucocorticoids in gastroprotective effect of RIPC. It is important to note that in our experimental conditioning we reproduced well known effect of PG deficiency on RIPC-caused gastroprotective effect: indomethacin pretreatment at non-ulcerogenic dose similar to metyrapone or RU-38486 also prevented the gastroprotective effect of RIPC.

In general, the natural gastric mucosal defensive mechanisms that counteract ulcerogenic stimuli are not completely elucidated, however a significant number of factors that participate in realization of these mechanisms were found; there are well known PGs, NO, glucocorticoids, heat shock proteins, trefoil peptides, growth factors, sensory innervation among them (Filaretova et al., 1998; Filaretova et al., 2002; Brzozowski et al., 2004a; Brzozowski et al., 2004b; Kobata et al., 2007; Filaretova, 2011; Gyires et al., 2015; Luetic et al., 2017). Most of these factors are possible participants of local IPC as well as RIPC-caused gastroprotection.

In most cases, I/R arise during trauma or surgery, when a temporary cessation or reduction of blood flow is inevitable. I/R represents a blood circulation recovery in ischemic organ or tissue which exacerbate the injury caused by ischemia by itself (Parks and Granger, 1988). I/R leads to an excessive production of reactive oxygen species and gastrin reducing simultaneously the local microcirculation provoking acute erosions and ulcer in gastric mucosa (Konturek et al., 2004).

Understanding of I/R pathogenesis and gastroprotective mechanisms as well as gastroprotective strategies is critical in the field of vascular surgery and pharmacology. Among such strategies special attention is paid to RIPC. RIPC represents a form of systemic protection against acute I/R injury and may provide non-invasive endogenous therapeutics strategy for protection organs against acute I/R (Kharbanda et al., 2002; Lim and Hausenloy, 2012; Heusch and Gersh, 2016). Although the phenomenon of RIPC has been known for a long time, its mechanisms are still partly unclear. Since glucocorticoid receptors are presented in almost all organs and tissues (Kanemasa et al., 1999), glucocorticoids could provide connection between preconditioned organ and target one. This connection can be provided not only by direct contact but also through the central effects like glucose level, blood pressure and vascular permeability or temperature homeostasis (Filaretova, 2011). Taking into consideration our previous findings demonstrating a compensatory gastroprotective role of glucocorticoids in the case of impaired gastroprotective mechanisms provided by PGs, NO, and capsaicin-sensitive sensory neurons (Filaretova et al., 2007) as well as the data on a participation of PGs, NO, and capsaicin-sensitive sensory neurons in realization of gastroprotective effect of RIPC (Brzozowski et al., 2004a; Brzozowski et al., 2004b) we assume a close interaction between these important gastroprotective factors in an implementation of RIPC-induced gastroprotection.

RIPC can exert the protective action against on I/R-induced injury in gastrointestinal tract through the reduction of inflammation (Zhu et al., 2021). Decrease of intestinal I/R induced damages after RIPC was accompanied by reduced mRNA expression of tumor necrosis factor α (TNF-α) and interleukin-6 (IL-6) (Zhu et al., 2021). Glucocorticoids, as anti-inflammatory drugs, inhibit the expression of multiple inflammatory genes including cytokines and, therefore, these hormones may provide the gastroprotective effect after RIPC through an inhibition of inflammation. A verification of this suggestion is the task for our future study.

Thus, the results of the present study demonstrate for the first time that non-invasive RIPC may protect the gastric mucosa against I/R-induced injury through involvement of glucocorticoids.

## Data Availability

The raw data supporting the conclusion of this article will be made available by the authors, without undue reservation.

## Abbreviations

CGRP, calcitonin gene-related peptide; CRF, corticotropin-releasing factor; I/R, ischemia-reperfusion; IPC, ischemic preconditioning; HPA axis, hypothalamic-pituitary-adrenocortical axis; PGs, prostaglandins; RIPC, remote ischemic preconditioning.
